# The repercussions of cyberbullying on students on educational platforms

**DOI:** 10.3389/fpsyg.2024.1426467

**Published:** 2024-11-07

**Authors:** Ghada Alturif, Hessa Alsand

**Affiliations:** Department of Social Planning, College of Social Work, Princess Nourah bint Abdulrahman University, Riyadh, Saudi Arabia

**Keywords:** bullying, cyberbullying, students, educational platforms, e-learning

## Abstract

The study aimed to explore the repercussions of cyberbullying on students on educational platforms. This descriptive study depends on a social survey for a sample of male and female students in their high school. The sample is 824 people, and data was collected through a questionnaire. The paper studies the repercussions of cyberbullying on students and the educational atmosphere. The study’s outcome is that most of the sample’s participants agree on these repercussions on both, students and the educational atmosphere. The study recommended holding conferences, webinars, and workshops, handling the repercussions of cyberbullying and regulations for bullies on educational platforms to deter this cyberbullying, to raise awareness among students.

## 1 Introduction

There is an increasing interest in cyberbullying through the internet on educational platforms. The reason behind this interest is increasing the number of cases of cyberbullying, specifically while using educational platforms a lot because of the COVID-19 epidemic. As a result, educational institutions depended on distance learning as a part of their educational system as one of the potentials to provide education on the Internet in all different majors by experts and specialized creative teachers.

Cyberbullying is one of the phenomena that became viral among educational circles through teenage students misusing electronic educational platforms which leads to hurdling the educational process and causing harm to students and teachers as well. Regarding what science has reached so far in the field of education, cyberbullying became a growing issue on these educational platforms, especially after accessing the internet became easier and became an integral part of students’ lives.

There is a difference between cyberbullying through the internet on educational websites and face-to-face bullying in schools. One of the most dominant differences between these two types of bullying is that cyberbullying last for a longer time and negatively affects individuals as well as it has various kinds and shapes which may cause harm to teenagers for a long time. Cyberbullying is more dangerous than other kinds of bullying as it depends on modern e-tools that, hugely, depend on the wide uncontrollable internet. The percentage of discovering the bully is very low as it is hard to directly face him/her.

The Ministry of Education in the KSA issued a digital behavioral guidebook on Madrasaty platform (my school) which aims to encourage and build positive behaviors among students for using verified electronic platforms in the KSA in public and private education. This guidebook also provides a disciplinary reference for students’ digital behaviors in using electronic platforms generally and in electronic learning especially. It also indicates the right behaviors for absolute benefit from electronic educational platforms in the KSA.

The guidebook is applied to all those who use educational platforms, including teachers, educational coordinators, school leaders, directors, students, and guardians. The guidebook includes the consequences that might be followed in case a violation happened on one of the educational platforms. Procedures would be followed according to the regulations of the guidebook (Saudi Ministry of Education, 2023).

This study discusses the cyberbullying of students on educational platforms to know the different shapes of bullying and its motives and focuses on knowing the repercussions of cyberbullying and the educational atmosphere as well on educational platforms. The answers of the participants may be from their previous experience or their witnessing of what happens to others in digital spaces in general and educational platforms in particular. Regarding that repercussion does not affect one part but also extend to affect all participants on educational platforms, such as students, teachers, teaching staff, trainers, and others participating in educational activities on the internet and educational platforms. That leads to psychological effects on students bullied by others which also causes hurdling the educational process. Therefore, it is important to study cyberbullying on educational platforms to restrict its negative effects, follow technical development, and benefit from its tools and function it to serve the educational process.

The growing development in the technology field is undoubtedly expanded its usage in different life areas, especially in the field of education which led to increasing the efficiency of the different shapes of e-learning and distance learning (Taybi, 2006). Adopting distance learning during the spread of pandemics and crises, and regarding the continuity of the educational process under the precautions of COVID-19 around the world led to using distance learning because of the ad-vantages achieved compared to the traditional ways that were impossible to use under the closure of the educational institutions (schools and universities) because of COVID-19 (Sayyed, 2021).

Therefore, distance learning became the main alternative for all educational stages in many countries around the world. Although it is important to impose distance learning whether in the pre or post-university stage and its advantages for the educational process, applying it showed a lot of disadvantages and limits, such as technical and behavioral problems. For example, cyberbullying affects the students and the educational atmosphere as well. Scientific pieces of research indicate that from 7 to 10 people were exposed to digital misusing one day (Abu Ghazaleh, 2018). Cyber-bullying is an extension of traditional bullying caused by social and psychological problems against those who have specific traits, such as handicaps, those who have difficulties in learning, and those who are different in shape, weight, color, etc. (Mahmoud, 2021).

Considering what education has reached so far on educational platforms because of COVID-19, cyberbullying became a growing problem on these educational platforms. Particularly, these platforms became easier to use and an integral part of daily students’ lives. That’s why we can see its wide-spreading between all stages of education starting from kindergarten until higher education. However, its danger is increasing among teenagers as they are facing changes in their shapes and psychologies which hugely affect their entire activities and behaviors. At this stage, their lives and academic future change in addition to the repercussions of bullying inside the virtual class. This makes teachers face challenges. School and teachers’ attitudes towards bullies have a huge effect on bullying rates (Raditch, 2019). A study indicates that 20% of teenage students are digitally bully or being bullied in one way or another (Mohammed, 2019a). As well as 35% of teaching members assure that they are bullied while practicing their jobs on educational platforms.

According to the behavioral studies that were conducted during the isolation period because of COVID-19, more than 1.5 billion children stayed in their houses which pushed them to use the internet to reach out to their lessons and hobbies. In other words, children got into the world of the internet at a younger age than usual and even without the minimum skills to protect themselves whether from harassment or cyberbullying as there are a lot of ways to humiliate and threaten kids through the internet. As a result, this causes psychological and social disorders (Al-Jizawi, 2021). So, we find it important to look into cyberbullying to know its motives and shapes on the education platforms as well as to know its repercussions on students and educational atmospheres. That helps handle the phenomena and restrict its negative impacts. The study’s problem is a question: What are the digital repercussions on students on educational platforms?

### 1.1 The importance of the study

1. This study is very important as cyberbullying is internationally, locally, and regionally a very significant topic because it has negative repercussions on individuals and communities.
2. The lack of Arabic and local studies that discussed the repercussions of cyberbullying on educational platforms. This research is focused to study the repercussions of cyberbullying on students and school environments on educational platforms.
3. This study enriches the Arab Library and provides a hypothetical and intellectual frame about cyberbullying cases in general and cyberbullying on educational platforms in particular.
4. The outcomes of this study benefit researchers, directors, and decision-makers to get to know the repercussions of cyberbullying on educational platforms which enables them to plan covering strategies and encourage being a digital citizen among educational platforms users.

This study aims to explore the repercussions of cyberbullying against students on educational platforms in an attempt to find solutions to restrict it.

### 1.2 Questions of the study

1. What are the social and economic characteristics of the respondents?
2. What are the cyberbullying forms on educational platforms?
3. What are the reasons behind increasing cyberbullying against students on educational platforms?
4. What are the repercussions of cyberbullying on students and educational atmospheres from the sample’s viewpoint?
5. Do the attitudes of the sample toward cyberbullying on educational platforms differ according to gender and monthly income?

## 2 Materials and methods

The current research adopts the social survey method as one of the primary approaches used in descriptive-analytical studies. This method is characterized by its ability to acquire a larger quantity of data and information within the constraints of time, effort, and available resources for the researchers. Consequently, it contributes to obtaining the necessary quantitative data to understand the reality and address research questions. Moreover, it facilitates description and goes beyond mere data description by striving to analyze the information and reach generalizable conclusions (Al-Tarif, 2019).

### 2.1 The definition of cyberbullying

Willard (2007) defines cyberbullying as, “sending or posting harmful or cruel text or images using the Internet or other digital communication devices”. Hinduja and Patchin (2008) defines it as, “willful and repeated harm inflicted through the use of computers, cell phones, and other electronic devices, such as computers and cell phones”. Tokunaga (2010) defines it as, “any behavior performed through electronic or digital media by individuals or groups that repeatedly communicates hostile or aggressive messages intended to inflict harm or discomfort on others”. Akbulut and Eristi (2011) defines it as, “the deliberate use of electronic communication tools through which harm or disturbance is intentionally and repeatedly delivered, targeting a specific individual or group of individuals”.

### 2.2 The definition of digital platforms

This is an interactive educational environment that employs web technology, combining the features of digital content management systems with social media net-works like Facebook and Twitter. It enables teachers to publish lessons, objectives, and assignments, as well as implement educational activities and communicate through various technologies. It facilitates the exchange of ideas and opinions between teachers and students, allowing for the sharing of educational content, ultimately leading to the achievement of high-quality educational outcomes (Assononi, 2019).

### 2.3 The theoretical framework

The impact of the COVID-19 pandemic on the education sector has been significant. Due to the widespread transmission of the coronavirus, many countries have shifted towards remote learning to protect students and teachers from the pandemic while ensuring the continuity of education. This global situation has compelled all nations to adopt and embrace remote learning as an indispensable alternative. Distance learning is a form of electronic education that bridges the physical gap between teachers and learners, allowing teachers to deliver content to students through communication technologies without the need for them to be physically present in the same location. Consequently, this educational system enables the dissemination of knowledge from distant and diverse locations to learners worldwide (Al-Akoul, 2021).

There are a lot of advantages to distance learning, such as contributing to raising cultural, scientific, and social levels among individuals, as well as, tackling the significant scarcity of educators and academic staff, strengthening the prospects of educational institutions, providing various educational sources for learners, and providing their expenses and transportations. Flexibility as well allows students to learn at the right time and place. It also gives the chance to get to know people from different backgrounds and nationalities. Additionally, distance learning is convenient as it is characterized to fit everybody. However, flexibility also gives the learner multiple choices to decide (Taybi, 2006).

On the other side, there is a number of disadvantages to distance learning. Some of these disadvantages are high costs for joining, not accepting communities to this kind of education, no interactivity, focusing the material on the theoretical part of the curriculum by cutting short live experiences and other benefits for the students, tiring learners as they spend long time on screens to follow their studies. Additionally, distance learning limits teachers’ roles only to education and neglects the educational side. Also, it disables students from evaluating their performance and doing their tasks. The appearance of some technical problems that might face both teachers and learners is another disadvantage of distance learning, such as losing internet connection, the lack of pieces of training on how to use the educational system, and students not having enough skills to use these modern technologies (Hassan, 2021).

Among all these disadvantages between students is cyberbullying which hurdle the educational process in distance learning by bullies doing unwanted negative and aggressive behaviors on educational platforms. This leads to limiting learners from learning and teacher from practicing their roles in making changes on students’ lives and achieving the goals of teaching (Al-Otaibi, 2021). A study by Jalisa (2022) indicates that students in distance learning face some challenges, such as affecting their social experiences and their relationships with each other. It is important to raise awareness to restrict spreading cyber-bullying. Saif (2021) stresses the importance of empowering teachers’ roles by using technologies correctly to achieve the ultimate goal of the educational process. This should be done shoulder to shoulder with their guardians and communities to guarantee that every kid will get the help they need. Al-Obeethani (2022) indicates the importance of developing the technical skills of teachers, students, and families to fight cyberbullying. Al-Jizawi (2021) insists on the significance of digital education, rising informational awareness, and being careful about virtual identities of families and their children.

### 2.4 Motives behind cyberbullying among students

There are plenty of Motives behind cyberbullying, and here are some:

#### 2.4.1 Psychological motives

It plays an essential role on forming bullies’ behaviors. These motives of bullies could be personal characteristics, such as physical strength, aggression, impulsiveness, lack of empathy, introversion, lack of confidence, and drawing attention (Rigby, 2007). Additionally, behavioral disorders, psychological diseases, depression, personality disorders, fury, addiction to aggressive behaviors, misunderstanding others, and worry affect humans’ behaviors and characters (Al-Tarif, 2012). Other researchers see that bullies feel unconfident, insecure, need to control and practice power over others. Feeling envy and jealous towards others’ potentials may lead them to practice bullying.

Karim (2020) indicates that the phenomenon of cyberbullying is a social problem that threatens the psychological well-being of individuals and communities. It has psychological consequences, such as feelings of frustration, isolation, and a desire to discontinue education, along with social and economic repercussions. Additionally, a study by Al-Qudayb (2020) confirms that cyberbullying leads to severe psychological and social problems, negatively impacting cognitive, social, and emotional development for both bullies and victims alike. These findings align with another study by Ben Salem (2020) which found various psychological effects on cyberbullying victims. Furthermore, Al-Jizawi (2021) suggests that one of the reasons behind bullying is a child’s desire to attract attention and portray themselves as strong to cope with personal frustrations.

#### 2.4.2 Social motives

Bullying may be attributed to disturbances in family relationships and the socioeconomic status of the family. Dysfunctional family structures, such as separation, divorce, continuous conflicts, or the absence of one parent from the family, can contribute to bullying behavior. Lack of awareness of cultural differences, ignorance of social up-bringing methods, repression, control, deprivation, and the absence of positive role models in parenting can also play a role (Amer, 2021). Exposure to physical abuse and harsh treatment within the home can lead children towards delinquent and deviant behavior (Patchin and Hinduja, 2006). Social relationship problems, increased feelings of loneliness, and fear of interacting with others are potential outcomes. Friends may also play a role in encouraging bullying as individuals often imitate their peers and follow in their footsteps. Additionally, a study by Al-Mustafa (2017) found that the motives for bullying can be linked to poor parental treatment, academic failure, and mistreatment by teachers, with differences in cyberbullying tendencies between males and females in favor of males. Furthermore, a study by Zaidi and Banai (2022) revealed numerous long-term negative effects of bullying, impacting social, psychological, and moral aspects. Bullying also supports violent behaviors, deviant practices, and perpetuates feelings of constant threat and anxiety.

#### 2.4.3 Economic motives

Economic conditions may contribute to making bullying happen as bullies may feel strong and powerful because of their economic situation. It could be completely the opposite as well because belonging to a poor layer of society and being in need of money could cause them to feel little, depressed, and weak, so they use bullying as a way of ex-pressing their feelings (Al-Mustafa, 2017). A study by Saad (2022) indicates that cyberbullying is attributed to eco-nomic, social, educational, and technological changes.

#### 2.4.4 School motives

This includes the type of environment, culture of the school, its administration, teaching quality, and the student’s relationship with teachers, as well as school safety and internet security. Social and environmental reasons: They include the education system, societal influence, cultural norms, student-peer relationships, cultural differences, and academic pressure (Al-Sarayreh, 2009).

The weakness of educational institutions in fulfilling their assigned role, such as tense relationships, authoritarian educational climate, huge numbers of students, crowd-ed classrooms, and challenging curricula, as well as punitive measures, dropouts, aca-demic failure, and disrupted relationships between students and teachers, and recurring absences, all these factors can push students towards bullying (Al-Kharji et al., 2021).

All these factors may lead students to frustration, which may drive them to engage in behavioral problems, some of which may manifest as direct or indirect bullying. Incorrect provocative practices by some teachers, the student’s low academic achievement, negative peer influence, moodiness, negligence by students, maladjustment or psychological issues, weak school-parent relationships, family and living conditions, teacher personality, authoritarian and discriminatory behavior towards students, and teachers’ lack of subject knowledge are all factors that may contribute to strengthening and displaying bullying behavior among some students (Al-Shahri, 2003). A study by Al-Mustafa (2017) indicated that the motivations for children’s bullying behavior are related to academic failure and mistreatment by teachers.

#### 2.4.5 Social and technological motives

Technical enhancements resulted in using social media by all people disregarding their ages. This also led to watching violent clips, horror movies, barbaric kills, and playing violent video games on computers or cell phones. All these practices encouraged viewers to copy what they watch in real life, in schools, acquaintances, or surroundings (Al-Ammar, 2016).

Saad (2022) indicates that cyberbullying depends on economic, social, educational, and technological circumstances. Al-Mustafa (2017) assures that bullies feel comfortable when harming others as they are having fun and showing their skills in using the computer.

### 2.5 Forms of cyberbullying among students on educational platforms

**Figure 1:**
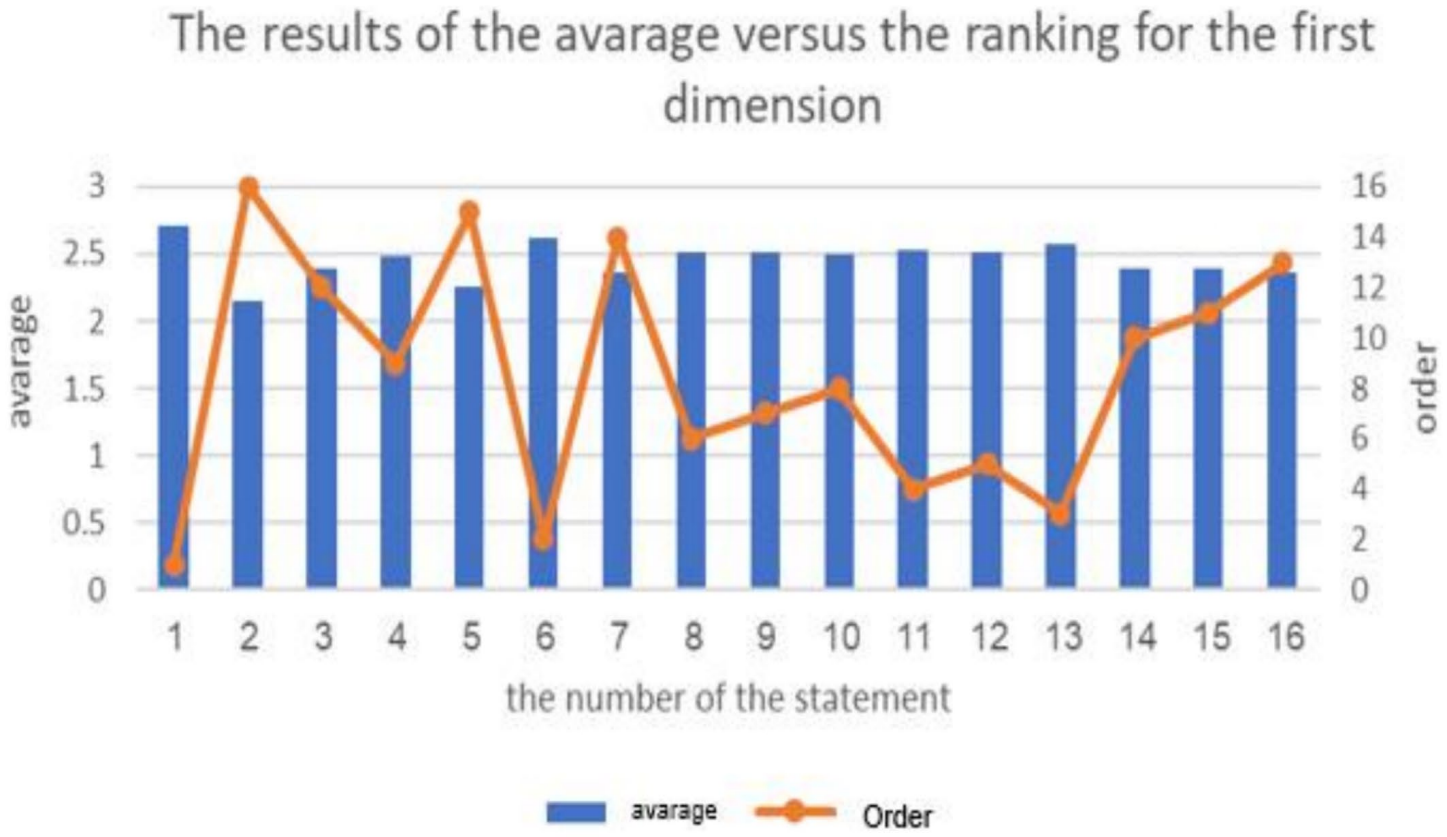
The results of the average the ranking forms of cyberbullying in educational platforms from students’ perspective.

**Figure 2:**
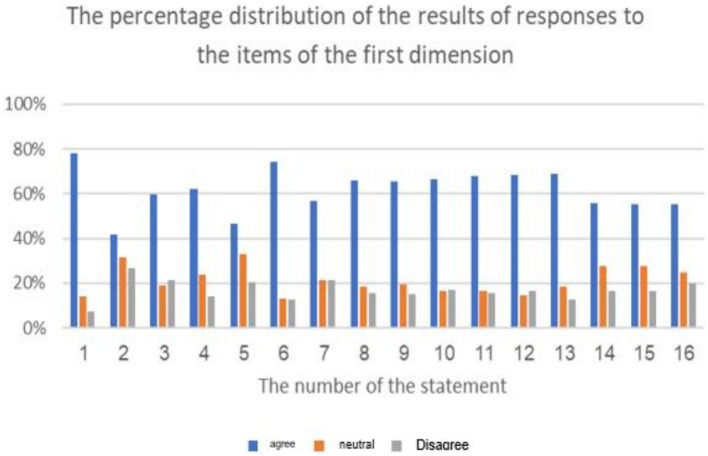
The percentage distribution of the forms of cyberbullying in educational platforms from students’ perspective.

**Figure 3:**
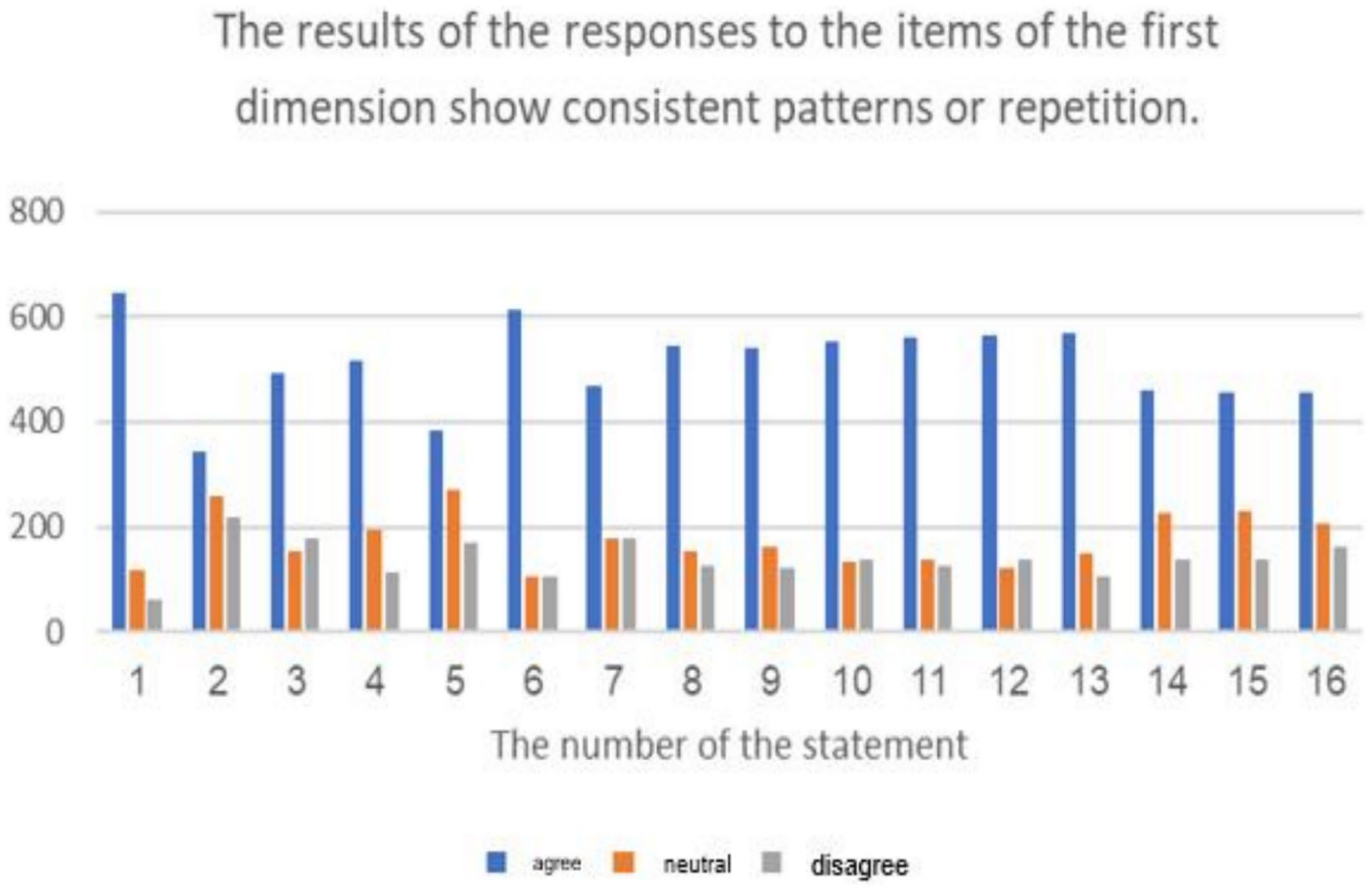
The results of the average the ranking for reasons behind increasing cyberbullying against students on educational platforms.

**Figure 4:**
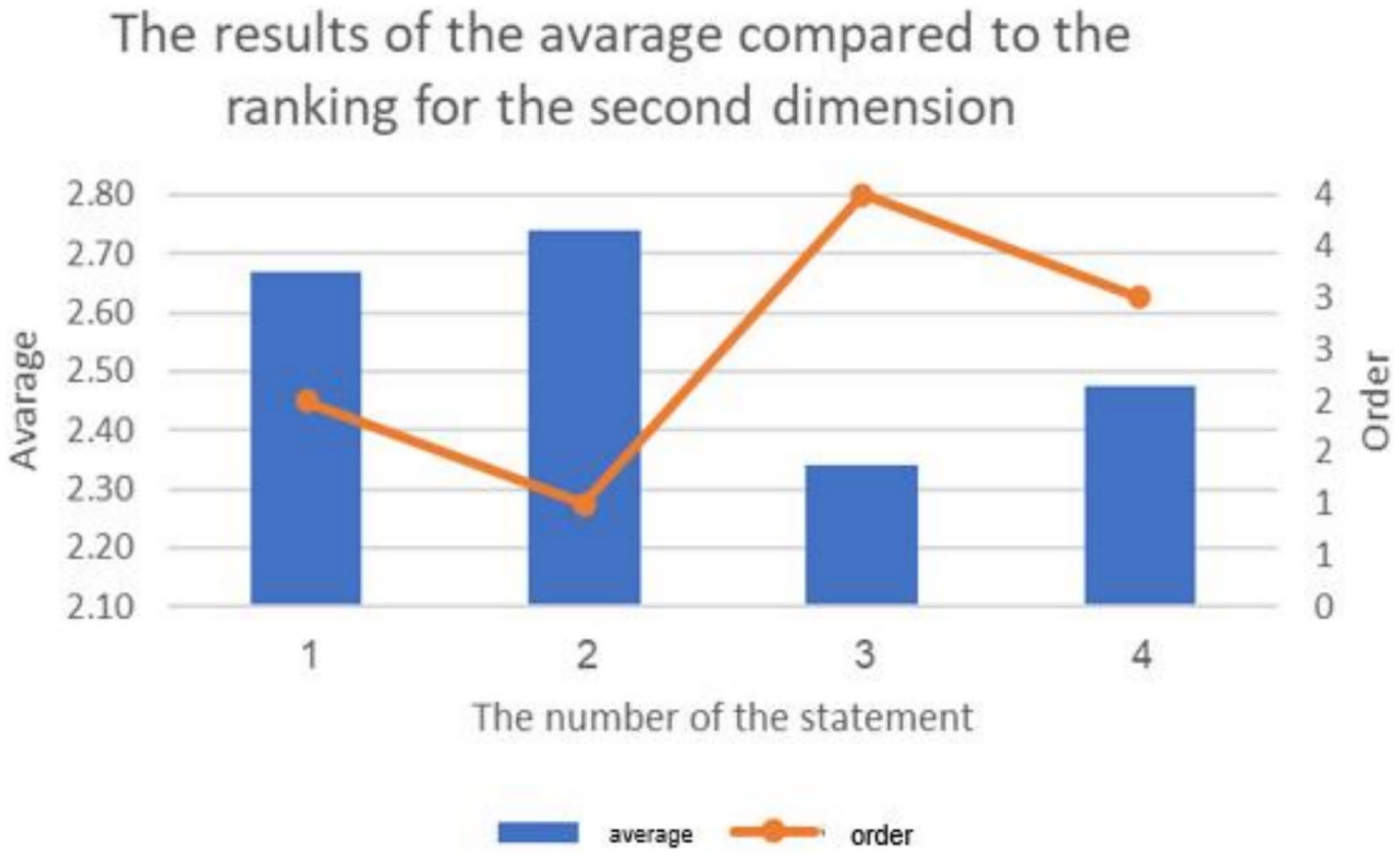
The results of the average the ranking for reasons behind increasing cyberbullying against students on educational platforms.

**Figure 5:**
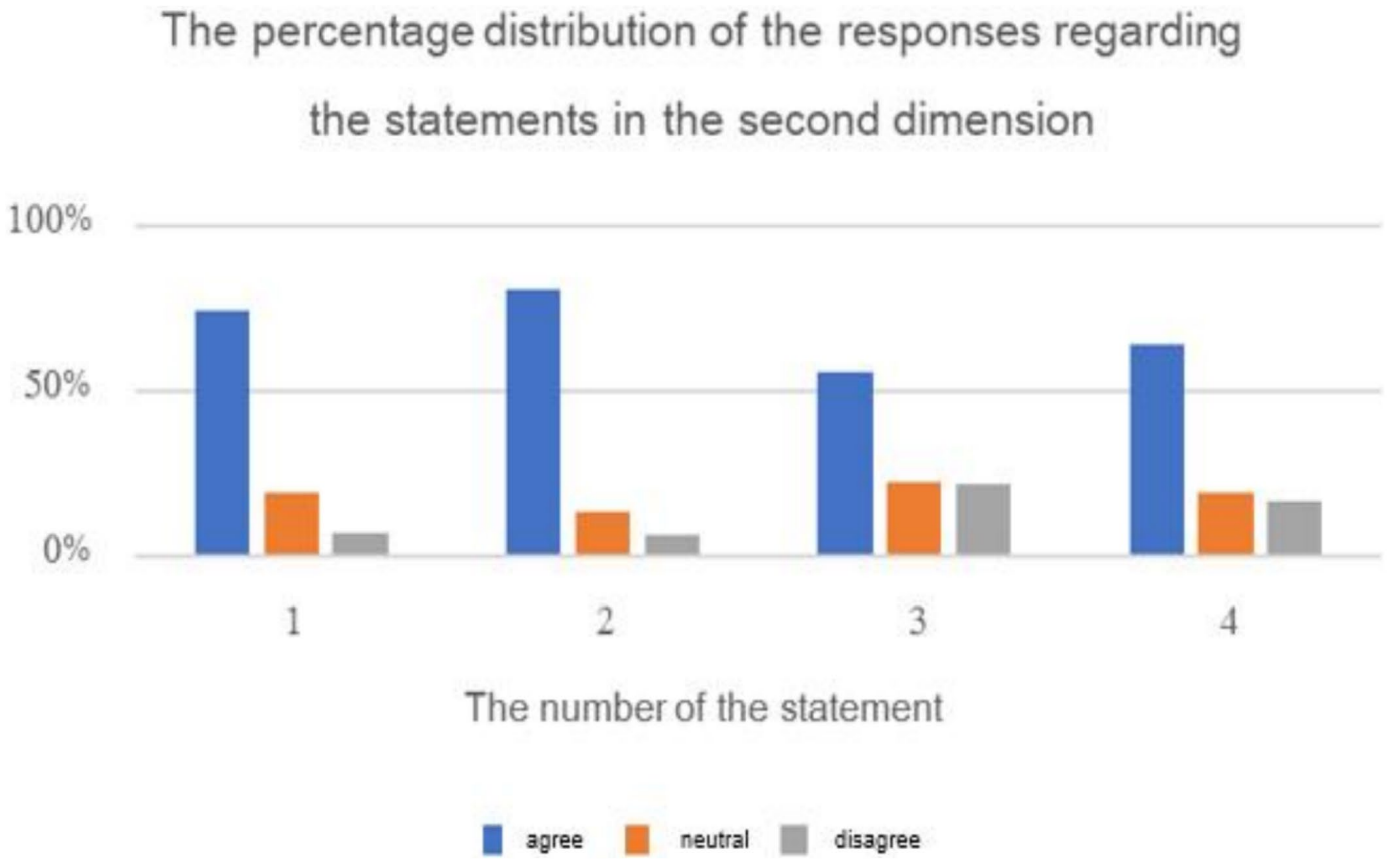
The percentage distribution of the reasons behind increasing cyberbullying against students on educational platforms.

**Figure 6:**
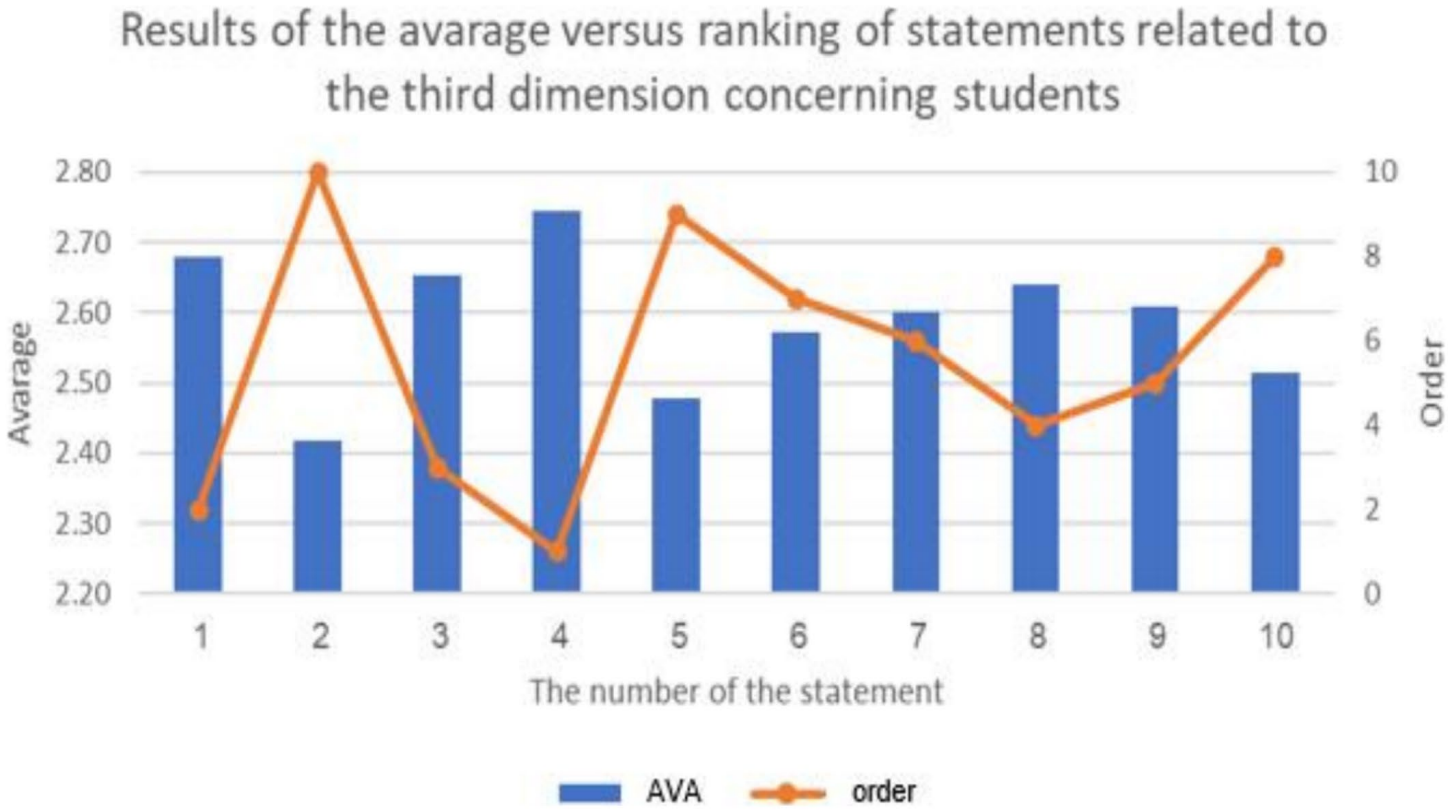
The results of the average of ranking the repercussions of cyberbullying on students.

**Figure 7:**
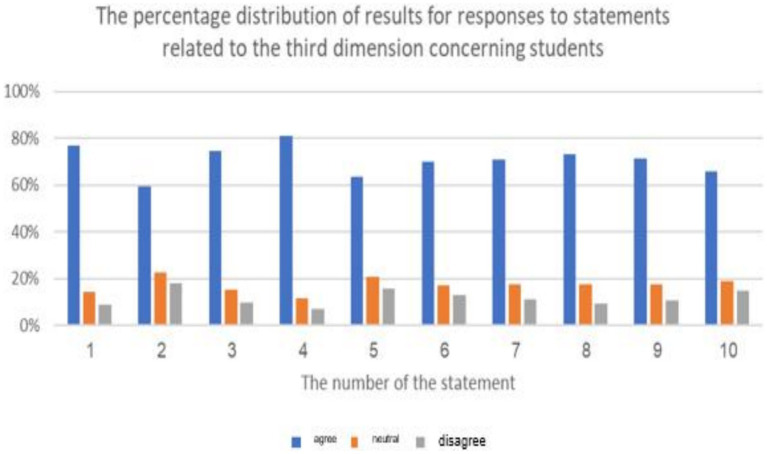
The percentage distribution of the repercussions of cyberbullying on students.

**Figure 8:**
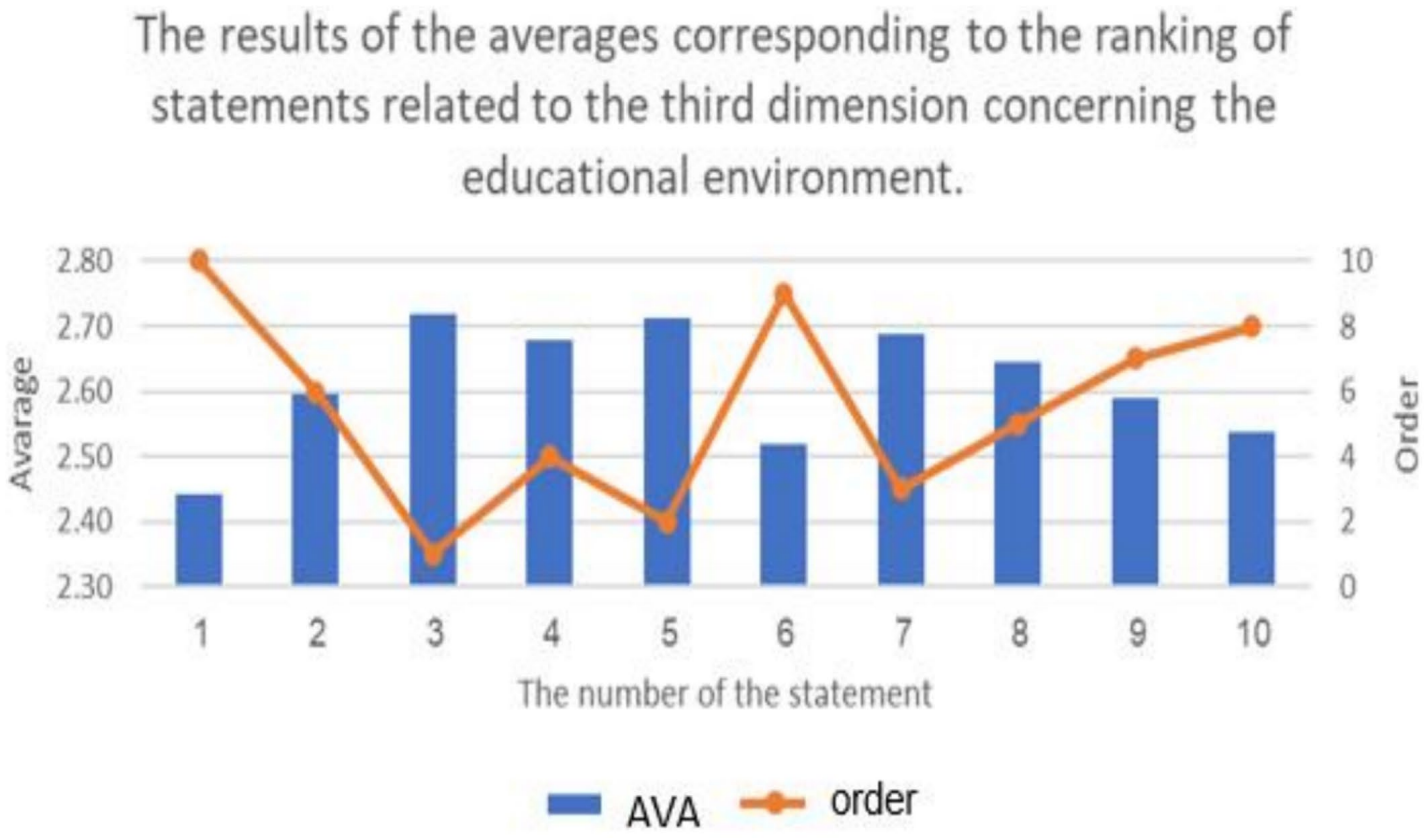
The results of the average corresponding to the ranking of statement related to repercussions of cyberbullying on educational atmospheres from the sample’s viewpoint.

**Figure 9:**
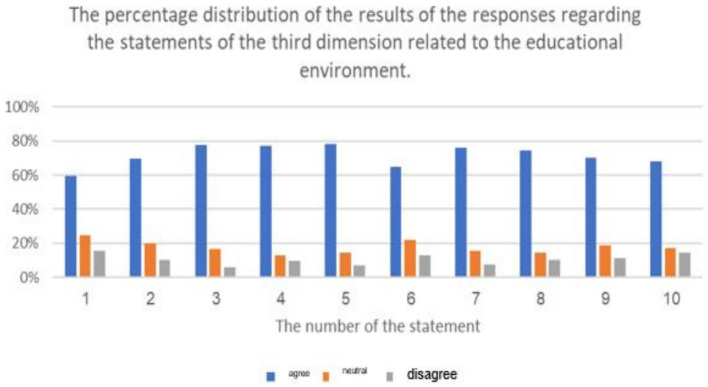
The percentage distribution of the results of the responses regarding the statements of the related to repercussions of cyberbullying on educational atmospheres from the samples’ viewpoint.

There are several forms of cyberbullying in educational platforms, including re-cording and sharing lessons without permission, filming and disseminating images of students without their knowledge, repeatedly interrupting intentional disruptions that impede the learning process and affect the targeted individual, altering the course of the lesson, sending mocking and aggressive messages based on appearance, academic weaknesses, or academic excellence, disabling or delaying student participation, and assuming the identity of others to hide the cyberbully’s true identity (see Figures 1–9; Tables 1–6).

**Table 1:** The distribution of the research sample members according to their social and economic characteristics, (824) individuals.

Variable	Sub-sets	Frequency	Percentage
Gender	Male	326	39.6%
	Female	498	60.4%
Monthly income rate	Low	94	11.4%
	Middle	568	68.9%
	High	162	19.7%

**Table 2:** Forms of cyberbullying in educational platforms from students’ perspective.

	Statement	Agree		Neutral		Disagree		Average	(χ2)	Order
		FRE	%	FRE	%	FRE	%			
1	Bullying is intentional, disturbing, and repetitive harassment or intimidation practiced by one student against another in order to control or harm them.	646	78%	116	14%	62	8%	2.71	758.3*	1
2	Recording the lesson and publishing it without prior permission from the lesson’s creator, who prepared it	344	42%	260	32%	220	27%	2.15	29.1*	16
3	Filming the students and publishing their pictures without their knowledge or consent	344	42%	260	32%	220	27%	2.39	263.6*	12
4	Repetitive targeted actions that hinder the learning process and negatively impact the person being bullied.	516	63%	194	24%	114	14%	2.49	329.7*	9
5	Changing the direction of the lesson by altering the subject or topic of the lesson.	384	47%	272	33%	168	20%	2.26	84.9*	15
6	Sending repeated aggressive mocking messages due to appearance, academic weaknesses, or academic excellence.	614	75%	106	13%	104	13%	2.62	628.8*	2
7	Disabling or delaying students’ participation.	470	57%	178	22%	176	21%	2.36	208.3*	14
8	Assuming another identity by concealing oneself to engage in bullying behavior.	546	66%	152	18%	126	15%	2.51	403.2*	6
9	Attempting to control the flow of the lesson, resulting in others remaining silent and not participating.	542	66%	160	19%	122	15%	2.51	392.9*	7
10	Spreading rumors within academic groups and chat groups that include all students of the class.	552	67%	134	16%	138	17%	2.50	420.1*	8
11	Exploiting others’ private data and sharing it without their consent and willingness.	562	68%	136	17%	126	15%	2.53	451.1*	4
12	Threatening with physical violence during an encounter.	566	69%	122	15%	136	17%	2.52	463.8*	5
13	Deceiving others to obtain their secrets or information.	570	69%	150	18%	104	13%	2.57	480.2*	3
14	Creating technical issues to delay the start of the lesson, muting others’ audio, or blocking them from accessing the educational platform.	460	56%	228	28%	136	17%	2.39	202.9*	10
15	Disrupting students’ focus and engagement in lessons and higher-order thinking processes.	458	56%	230	28%	136	17%	2.39	199.6*	11
16	Tampering with the presentation slides of the lesson or drawing on them.	456	55%	206	25%	162	20%	2.36	183.1*	13

Significant (*≥ 0.01).

**Table 3:** The reasons behind increasing cyberbullying against students on educational platforms.

	Statement	Agree		Neutral		Disagree		Average	(χ2)	Order
		FRE	%	FRE	%	FRE	%			
1	Social reasons, weak parental supervision, and family disintegration.	610	74%	156	19%	58	7%	2.67	631.5*	2
2	Psychological reasons, such as mental disorders, jealousy, the desire to seek attention from others, and boredom.	662	80%	108	13%	54	7%	2.74	824.6*	1
3	Economic reasons, such as high-or low-income levels, financial deprivation, and rising prices.	460	56%	186	23%	178	22%	2.34	187.7*	4
4	Technological factors, such as watching violent actions in movies and games and imitating scenes of violence and harm	528	64%	158	19%	138	17%	2.47	351.2*	**3**

Significant (*≥ 0.01).

**Table 4:** Consequences of cyberbullying on students and the learning environment in educational platforms.

	Statement	Agree		Neutral		Disagree		Average	(χ2)	Order
		FRE	%	FRE	%	FRE	%			
The repercussions of cyberbullying on students on educational platforms										
1	Psychological disorders such as depression, anxiety, and cyberphobia.	634	77%	116	14%	74	9%	2.68	708.3*	2
2	Exposure to various physical illnesses.	492	60%	184	22%	148	18%	2.42	260.3*	10
3	Feelings of isolation, loneliness, and introversion.	618	75%	126	15%	80	10%	2.65	647.6*	3
4	Lack of confidence in oneself and others.	672	82%	94	11%	58	7%	2.75	864.5*	1
5	Suicidal thoughts and the desire to harm oneself.	524	64%	170	21%	130	16%	2.48	342.4*	9
6	Resorting to violence for self-defense.	578	70%	140	17%	106	13%	2.57	504.5*	7
7	Academic decline and school avoidance.	586	71%	148	18%	90	11%	2.60	535.4*	6
8	Social adaptation difficulties.	604	73%	144	17%	76	9%	2.64	600.7*	4
9	Engaging in continuous fights.	590	72%	146	18%	88	11%	2.61	549.1*	5
10	Avoiding participation in school activities.	546	66%	156	19%	122	15%	2.51	404.1*	8
The repercussions of cyberbullying on the school environment in educational platforms										
1	General decline in the performance of the education system.	492	60%	204	25%	128	16%	2.44	268.4*	10
2	Impact on the educational relationship between teachers and students.	576	70%	162	20%	86	10%	2.59	506.3*	6
3	Loss of respect for others and the absence of a culture of dialogue among students.	640	78%	136	17%	48	6%	2.72	742.9*	1
4	The absence of psychological safety and well-being in the learning environment.	638	77%	108	13%	78	9%	2.68	722.5*	4
5	The transition of cyberbullying among students from online learning platforms to real-life situations.	644	78%	122	15%	58	7%	2.71	752.3*	2
6	The students’ failure to benefit from educational programs and training provided to them.	536	65%	180	22%	108	13%	2.52	382.4*	9
7	The prevalence of aggressive and violent behaviors in the school environment.	630	76%	130	16%	64	8%	2.69	697.4*	3
8	The frequent occurrence of behavioral violations and the disregard of school rules and regulations.	618	75%	120	15%	86	10%	2.65	645.8*	5
9	Vandalism of public property in the educational environment.	578	70%	154	19%	92	11%	2.59	509.4*	7
10	The school’s bad reputation.	562	68%	142	17%	120	15%	2.54	451.7*	8

Significant (*≥ 0.01).

**Table 5:** Results of the independent samples test to examine the difference in participants’ attitudes towards cyberbullying based on gender.

Gender	Number	Dependent variables	Average	Standard deviation	Degrees of freedom	Value of (*T*)	Level of significance
Males	326	Causes of cyberbullying in educational platforms from the students’ perspective	2.556	0.710	822	−0.795	0.525
Females	498		2.555	0.711			
Males	326	Forms of cyberbullying in educational platforms from the students’ perspective	2.453	0.763	822	0.006	0.478
Females	498		2.453	0.763			
Males	326	The repercussions of cyberbullying on students on educational platforms	2.592	0.690	822	0.456	0.484
Females	498		2.591	0.691			
Males	326	The repercussions of cyberbullying on the school environment on educational platforms	2.611	0.669	822	0.467	0.413

**Table 6:** One-way ANOVA results to examine the differences in participants’ attitudes towards cyberbullying based on monthly income.

Monthly income	Number	Dependent variables	Average	standard deviation	Degrees of freedom	Value of (*T*)	level of significance
Low	94	Causes of cyberbullying in educational platforms from the students’ perspective	2.552	0.712	728	−0.215	0.509
Middle	568		2.555	0.711			
High	162		2.554	0.711			
Low	94	Forms of cyberbullying in educational platforms from the students’ perspective	2.448	0.765	728	0.756	0.414
Middle	568		2.543	0.763			
High	162		2.448	0.764			
Low	94	The repercussions of cyberbullying on students on educational platforms	2.588	0.692	728	−0.448	0.492
Middle	568		2.591	0.691			
High	162		2.588	0.691			
Low	94	The repercussions of cyberbullying on the school environment on educational platforms	2.606	0.672	728	−0.0790	0.424
Middle	568		2.612	0.669			
High	162		2.606	0.672			

Additionally, attempts to control the flow of the lesson can lead to silencing others and preventing their participation. Cyberbullies spread rumors in groups and academic communities that include all students in the class. They exploit others’ private data and share it without their consent, threatening physical violence during online meetings, deceiving others to obtain their secrets or information, creating technical problems to delay attendance, mute or block others from the educational platform, disrupting the concentration and focus of students on lessons and higher-order thinking, and tampering with les-son slides or drawing on them (Khalifa et al., 2020). A study by Al-Jizawi (2021), highlights various forms of cyberbullying, including cyberbullying through jokes, isolating and marginalizing the victim by excluding them from online activities or groups, and uploading embarrassing photos that harm the victim’s reputation. Another study by Ben Dada and Karim (2021), indicates that the most prevalent and recurring forms of cyberbullying are exclusion and sexual harassment, followed by annoyance, privacy violations, insults, threats, and finally, mocking and defamation of character. A study by Mohammed (2019b), points out that prominent forms of cyberbullying include ridicule, defamation of others, spreading rumors, posting disturbing images, harassment, repeated insults in all forms, identity impersonation or theft to embarrass or destroy someone and harm their reputation, exposing secrets, and electronic stalking and harassment. A study by Zaid (2020), confirms that the most prominent forms of cyberbullying faced by teenagers include sharing personal secrets, imposing opinions and beliefs, followed by luring them into engaging in inappropriate behavior and then threatening to expose it. Additionally, exploiting personal photos and videos available on digital media for malicious purposes and sharing them, followed by sharing inappropriate content, accessing personal accounts and sharing private matters through digital media, and finally receiving inappropriate text messages from strangers.

### 2.6 Repercussions of cyberbullying on educational platforms

When discussing the repercussions of cyberbullying on educational platforms, we find that they do not affect one party alone but extend to impact all participants on the educational platform. It affects students in general and the victims in particular, as well as teachers and faculty members, and all participants in the educational platforms.

Regarding the repercussions of cyberbullying on educational platforms on students in general and victims in particular, it has been observed that they face difficulties in interacting with the educational content and other students in an interactive learning community through these platforms (Tomczyk and Wloch, 2019). The issues caused by cyberbullying students in training courses, educational programs, and lessons on educational platforms have detrimental effects on students. These effects include disrupting the focus and higher-order thinking processes, and may even lead to a situation where a student bullies a more diligent peer, obstructing others from listening to them, muting or blocking their voice, and even excluding them from the educational platform. All these various forms of cyberbullying can lead to psychological harm for the targeted students, hinder the educational process, lower academic performance, and result in a decreased participation in educational tasks provided through these platforms (Mohammed, 2019b).

Several studies have emphasized the importance of facing cyberbullying to mitigate its negative effects. Studies including Al-Brashidiya (2020), Al-Obeethani (2022), and Ben Dada and Karim (2021), recommend the implementation of preventive and training programs to address this phenomenon, as it can have serious psychological and social implications on individuals and society. A study by Qutb (2022), high-lights the importance of raising awareness and familiarizing individuals with the legal consequences under cybercrime laws. Moreover, a study by Sayyed (2020), points out various intervention approaches to combat cyberbullying, including digital security, fostering religious awareness, family support, enforcing digital laws, promoting mental well-being, providing media support, and offering peer support.

## 3 Methodology

### 3.1 Research type

This study belongs to the category of descriptive-analytical research, which aims to present data and facts about a specific phenomenon or subject in a precise and detailed manner. It also explores the relationship between various phenomena and their correlation with the phenomenon itself, aiding in predicting its future outcomes. The research employed quantitative methods (questionnaires) to collect and describe the data in a numerical format, indicating the quantity or magnitude of the phenomenon. Subsequently, it arrived at conclusions, generalizations, and new relationships based on the findings (Pandey, 2014).

The study aims to explore the causes, forms, and consequences of cyberbullying on students and the school environment within educational platforms.

### 3.2 Research methodology

The current research adopts the social survey method as one of the primary approaches used in descriptive-analytical studies. This method is characterized by its ability to acquire a larger quantity of data and information within the constraints of time, effort, and available resources for the researchers. Consequently, it contributes to obtaining the necessary quantitative data to understand the reality and address research questions. Moreover, it facilitates description and goes beyond mere data description by striving to analyze the information and reach generalizable conclusions (Al-Tarif, 2019).

### 3.3 Research community

The research community consists of a random sample of male and female high school students from several key regions in the Kingdom of Saudi Arabia (Northern region, Southern region, Eastern region, Western region, and Central region).

### 3.4 Sample of the study

In descriptive research, the scientific investigation focuses on a scientific phenomenon that arises from a large population. It is not feasible for the researcher to study the entire population, so a representative sample is chosen from it (Abdelmoneim, 2012). To determine a representative sample, the Kingdom’s regions were divided into five main areas: Northern, Southern, Eastern, Western, and Central regions. These areas were considered as strata in the first stage (stratified sampling). In the second stage, each educational administration in the same region was treated as clusters (cluster sampling). In the third stage, a random sample of high schools was selected from each region (simple random sampling). Finally, a random sample was drawn from each cluster, which means a random sample of students of both genders in the schools, using simple random sampling. This process ensures a high level of randomness in the selection and representation of the chosen sample of students across different regions and districts (Al-Maaytah, 2011). Consequently, a random sample of male and female students from high schools nationwide was selected, totaling 100 government secondary schools distributed across the Kingdom. Then, 20 schools were randomly chosen to represent different regions of the Kingdom. The total sample size was 824 respondents.

### 3.5 Study tools

The questionnaire was selected as the primary tool for collecting field data, considering the nature of the subject and the study’s objectives. The questionnaire is deemed suitable for obtaining information, data, and facts related to a specific reality. It consists of a set of questions that individuals within the study community are requested to answer (Sabir, 2003). The questionnaire was prepared based on the theoretical framework and previous studies in this field. It includes preliminary data related to gender and monthly income. The questionnaire consists of 40 statements that measure three main dimensions: forms of cyberbullying among students in educational platforms (16 statements), causes of cyber-bullying among students in educational platforms (4 statements), and repercussions of cyberbullying. The consequences of cyberbullying are distributed across two domains: its effects on students in educational platforms (10 statements) and its impact on the school environment in educational platforms (10 statements).

The responses to the questionnaire statements were collected using a three-point Likert scale, with the following three response options: (agree, neutral, disagree). These three responses were assigned scores of (3, 2, 1) respectively, to assess the reliability and validity of the questionnaire. The questionnaire was administered to a sample of (70) male and female students. The reliability and validity of the questionnaire were calculated as follows:

#### 3.5.1 First: reliability of the questionnaire

The reliability of the statements in the questionnaire on the repercussions of cyberbullying on students on educational platforms was calculated using the Cronbach’s alpha test with a reliability level of 99%. The obtained reliability coefficient was 95.4%, indicating the accuracy and consistency of the responses. The questionnaire received (824) valid responses suitable for statistical analysis out of (40) different questions across three main dimensions.

#### 3.5.2 Second: validity of the questionnaire

Face Validity: The questionnaire was presented to a group of specialized professors in the Department of Sociology and Social Services at Princess Nourah bint Abdulrahman Uni-versity and Imam Muhammad Ibn Saud Islamic University. Their input was sought to assess the appropriateness, importance, clarity, and formulation of the questionnaire items. Based on their feedback, the questionnaire was modified and prepared in its final form.

### 3.6 Calculating the validity of the questionnaire statements

An alpha Cronbach analysis was conducted for all the questions included in the questionnaire across the four dimensions, totaling (40) questions. The result was 95.5%, indicating a high level of internal consistency among the questionnaire items. This also suggests that the questionnaire exhibits a high degree of validity.

#### 3.6.1 The statistical methods used for data analysis

To achieve the study objectives and analyze the collected data, various appropriate statistical methods were used, including the calculation of the arithmetic mean, standard deviation, and frequency analysis. Additionally, the Chi-Square (χ2) test was applied to explore differences in response frequencies among the sample individuals across all the items in the study tool. The Independent Samples Test (t) was utilized to investigate differences between social and economic variables in the study sample. Furthermore, the One-Way ANOVA (analysis of variance) was employed for one-directional analysis.

## 4 Results

### 4.1 First question: What are the social and economic characteristics of the respondents?

The above table illustrates the distribution of the research sample members ac-cording to their social and economic characteristics. The results indicate that the majority of the sample members are females, comprising 60%, while males make up 39.6%. Regarding economic status, the majority fall under the middle-income category, accounting for 68.9%, followed by those with high income at 19.7%. The lowest proportion was ob-served among individuals with low income, constituting 11.4%.

### 4.2 Second question: What are the cyberbullying forms on educational platforms?

The above table indicates the presence of statistically significant differences (at the 0.01 ≥ α level) in the response frequencies among the sample individuals in favor of the “Agree” response on all items of the first dimension: “Forms of Cyberbullying in Educational Platforms from Students’ Perspective.” The average scores for the items in this dimension ranged from 2.15 to 2.71, and all of these averages fall within the “Agree” response range (which extends from 2.33 to 3), except for the second and fifth items, where the response was “Neutral.” These responses fall within the “Neutral” response range (ranging from 1.66 to 2.33) for the items “Recording the lesson and publishing it without prior permission from the lesson’s creator, who prepared it” and “Changing the direction of the lesson by altering the subject or topic of the lesson,” respectively. In summary, the majority of the sample individuals statistically agree with the fourteen methods of cyber-bullying in educational platforms.

The highest average for items of the dimension “Forms of Cyberbullying in Educational Platforms from Students’ Perspective” reached (2.71 out of 3) from the sample’s point of view. The cyberbullying method “Harassment practiced by a student against an-other deliberately, disturbingly, and repeatedly to control or harm them” had the highest average. Meanwhile, the lowest average for items in this dimension was (2.15), which was attributed to the cyberbullying method of “Recording the lesson and publishing it without prior permission from the lesson’s creator, who prepared it.” The study’s findings align with previous research on cyberbullying, such as Mohammed (2019a), Al-Jizawi (2021), Ben Dada and Karim (2021), Khalifa et al. (2020), and Zaid (2020), all of which emphasize the various forms of cyberbullying that students experience on educational platforms.

### 4.3 Third question: What are the reasons behind increasing cyberbullying against students on educational platforms?

The previous table reveals statistically significant differences (at the 0.01 ≥ α level) in response frequencies among the sample individuals in favor of the “Agree” response on all items of the second dimension: “Causes of Cyberbullying in Educational Platforms from Students’ Perspective.” This indicates that the majority of the sample individuals statistically agree with all the reasons for cyberbullying on educational platforms. The aver-age scores for the items in this dimension ranged from 2.34 to 2.74, and all of these aver-ages fall within the “Agree” response range (which extends from 2.33 to 3).

The results reveal that the highest average for items in the dimension “Causes of Cyberbullying in Educational Platforms from Students’ Perspective” from the sample’s perspective was (2.74 out of 3). The psychological reasons for cyberbullying had the highest average (2.67), followed by social reasons (2.47). The lowest average for items in this dimension was (2.34) for economic reasons. The findings align with previous studies that investigated psychological reasons for cyberbullying, such as studies by Al-Jizawi (2021), Al-Qudayb (2020), Ben Salem (2020), and Karim (2020), which confirmed that cyberbullying is related to personality disorders and has various psychological effects on the victims. The study’s results also correspond with studies exploring social causes of cyberbullying, such as studies by Amer (2021), Patchin and Hinduja (2006), Tomczyk and Wloch (2019), and Zaidi and Banai (2022), which indicated that cyberbullying has multiple social reasons. Furthermore, the study’s findings align with previous research on economic causes of cyberbullying, such as studies by Al-Mustafa (2017) and Saad (2022), which suggest that economic conditions play a role in cyber-bullying. The cyberbully may feel empowered and in control due to their higher economic status, while conversely, belonging to a lower socio-economic class and facing financial needs may lead to feelings of inadequacy and frustration, prompting them to engage in cyberbullying as a means of venting their emotions. Additionally, the study’s findings are consistent with research exploring media and technological causes of cyberbullying, such as studies by Al-Mustafa (2017), Al-Shahri (2003), and Saad (2022), which suggest that cyberbullies feel comfortable inflicting harm on others to entertain themselves and showcase their computer skills.

### 4.4 Forth question: What are the repercussions of cyberbullying on students and educational atmospheres from the sample’s viewpoint?

The previous table shows statistically significant differences (at a level of 0.01 ≥ α) in the response frequencies of the sample individuals in favor of the response (agree) for all statements related to the third dimension: (the repercussions of cyberbullying on students and the educational environment in e-learning platforms). This indicates that the highest percentage of the sample individuals statistically agree with all the repercussions of cyberbullying on both students and the educational environment in e-learning platforms.

It is evident that the averages of statements related to the third dimension, concerning the domain of (the repercussions of cyberbullying on students on educational platforms), ranged from (2.42) to (2.75), and all these averages fall within the range of response (agree), confirming the agreement of the sample individuals with all the repercussions of cyberbullying on students on educational platforms. The highest average for the domain (the repercussions of cyberbullying on students in e-learning platforms) from the perspective of the sample was (2.75 out of 3), and it was for the statement (lack of self-confidence in oneself and others), while the lowest average for statements in this do-main was (2.42), and it was for the statement (exposure various physical illnesses).

Additionally, the averages of statements related to the third dimension, concerning the domain of (the repercussions of cyberbullying on the educational environment on educational platforms), ranged from (2.44) to (2.72), and all these averages fall within the range of response (agree), confirming the agreement of the sample individuals with all the repercussions of cyberbullying on the educational environment. The highest average for the domain (the repercussions of cyberbullying on the educational environment on educational platforms) from the perspective of the sample was (2.72 out of 3), and it was for the statement (lack of respect for others and absence of a culture of dialogue among students), while the lowest average for statements in this domain was (2.44), and it was for the statement (decline in the overall performance of the educational system). The results of the study align with the findings of previous studies such as Al-Jizawi (2021), Karim (2020), and Saad (2022). Due to the negative repercussions of cyberbullying, several studies have emphasized the importance of confronting cyberbullying to mitigate its negative effects, including studies conducted by Al-Brashidiya (2020), Al-Obeethani (2022), Ben Dada and Karim (2021), Karim (2020), Qutb (2022), and Sayyed (2020).

### 4.5 Fifth question: Do the attitudes of the sample toward cyberbullying on educational platforms differ according to gender and monthly income?

The previous table indicates that there were no statistically significant differences in each of the following dimensions: (causes of cyberbullying on educational platforms from the students’ perspective), (forms of cyberbullying on educational platforms from the students’ perspective), (repercussions of cyberbullying on students on educational plat-forms), and (repercussions of cyberbullying on the school environment on educational platforms). These findings are attributed to the monthly income level; as the values of (*T*) were not statistically significant, suggesting that there is no variation in the participants’ attitudes towards cyberbullying concerning its causes, methods, and different consequences on students and the educational environment based on income level.

## 5 Conclusion

The study aimed to investigate the repercussions of cyberbullying on students on educational platforms. The study sample consisted of 824 male and female high school students, and data were collected through a questionnaire. The study yielded the following results:

1. Regarding the forms of cyberbullying on students on educational platforms, the study found that the highest percentage of the sample statistically agreed on all forms of cyber-bullying on students on educational platforms. The highest percentage was for the form of cyberbullying characterized by “Intentional, disturbing, and repetitive harassment of a student by another to control or harm them.” On the other hand, the lowest mean was for the items related to “Taking and sharing photos of a lesson without prior permission from the lesson’s author,” and “Altering the course of a lesson by changing its topic”.
2. Regarding the reasons for cyberbullying on students in educational platforms, the study found that the highest percentage of the sample statistically agreed on all reasons for cyberbullying on students on educational platforms. The order of agreement was as follows: psychological reasons ranked highest, followed by social reasons, and then reasons related to technological advancements. The lowest mean was for economic reasons.
3. Regarding the repercussions of cyberbullying on students on educational platforms, the study’s results showed that the highest percentage of the sample statistically agreed on all consequences of cyberbullying on both students and the school environment in educational platforms. The highest mean for the domain of “Repercussions of cyberbullying on students on educational platforms” from the participants’ perspective was related to “Lack of oneself confidence and others,” while the lowest mean was related to “Exposure to various physical illnesses.” As for the repercussions of cyberbullying on the educational environment on educational platforms, the highest mean was related to “Loss of respect for others and absence of a culture of dialogue among students,” while the lowest mean was related to “General decline in the performance of the educational system”.
4. Regarding the researchers’ attitudes towards cyberbullying based on gender and monthly income, the study’s results indicated that there were no statistically significant differences in all of the following aspects: forms of cyberbullying on educational platforms, reasons for cyberbullying on educational platforms, and repercussions of cyberbullying on students and the school environment on educational platforms, in relation to gender and monthly income variables. This indicates that there were no differences in the researchers’ attitudes towards cyberbullying in terms of its forms, reasons, and various repercussions on students and the educational environment, based on the gender and monthly income variables in the study.

## Data Availability

The original contributions presented in the study are included in the article/supplementary material, further inquiries can be directed to the corresponding author.
